# Significant evidence for a heritable contribution to cancer predisposition: a review of cancer familiality by site

**DOI:** 10.1186/1471-2407-12-138

**Published:** 2012-04-03

**Authors:** Frederick Albright, Craig Teerlink, Theresa L Werner, Lisa A Cannon-Albright

**Affiliations:** 1 Department of Pharmacotherapy, University of Utah College of Pharmacy, Salt Lake City, UT, USA; 2 Genetic Epidemiology, Department of Medicine, University of Utah School of Medicine, Salt Lake City, UT, USA; 3 Oncology, Department of Medicine, University of Utah School of Medicine, Salt Lake City, UT, USA; 4 George E. Wahlen Department of Veterans Affairs Medical Center, Salt Lake City, UT, USA

## Abstract

**Background/Aims:**

Sound and rigorous well-established, and newly extended, methods for genetic epidemiological analysis were used to analyze population evidence for genetic contributions to risk for numerous common cancer sites in Utah. The Utah Population Database (UPDB) has provided important illumination of the familial contribution to cancer risk by cancer site.

**Methods:**

With over 15 years of new cancer data since the previous comprehensive familial cancer analysis, we tested for excess familial clustering using an expanded Genealogical Index of Familiality (dGIF) methodology that provides for a more informative, but conservative test for the existence of a genetic contribution to familial relatedness in cancer.

**Results:**

Some new cancer sites have been analyzed for the first time, having achieved sufficiently large sample size with additions to the UPDB. This new analysis has identified 6 cancer sites with significant evidence for a heritable contribution to risk, including lip, chronic lymphocytic leukemia, thyroid, lung, prostate, and melanoma.

**Conclusions:**

Both environmentally and genetically-based familial clustering have clinical significance, and these results support increased surveillance for cancer of the same sites among close relatives of affected individuals for many more cancers than are typically considered.

## Background

The data for this study comes from a well known and scientifically recognized computerized resource in Utah, the Utah Population Database (UPDB) [1]. The UPDB was derived from the computerization of genealogical data for the Utah pioneers and their descendants. Originally it was used as a data resource to study the heritability of cancer risk [2-6]. Over the decades of its existence the UPDB has evolved to include general population data, as well as more recent genealogy created from Utah State Vital Records (such as mother, father, child triplets from birth certificates). The UPDB has been record linked to state death certificates as well as to diagnosis data from the largest Utah Hospitals and clinics.

The Utah Cancer Registry (UCR) was established in 1973 and is one of the earliest member state registries for the Surveillance, Epidemiology, and End Results (SEER) Program of the National Cancer Institute (NCI) [7-9]. The individual data in the UPDB continues to be linked annually to the statewide cancer diagnosis data of the UCR [10]. The UCR has recorded cancers in the state since 1966. The combination of genealogy data in the UPDB with individual cancer data in the UCR allows genetic epidemiological analysis of familial clustering of cancer by site.

The most recent published report on familial clustering of cancer by site in Utah was in 1994 [3]. This current study gives an update of evidence for familial clustering of cancer in Utah, and includes an enhanced analysis of familiality methodology that allows a more detailed examination of the *heritable contribution *to observed familial clustering. This current analysis of the latest UPDB linked data has provided larger sample sizes for all sites previously reported, and includes some new sites which have gained sufficient sample sizes since 1994 to be analyzed for the first time.

## Methods and data resources

### Utah population database

The Utah genealogy data was originally linked to Utah cancer registry records in the late seventies. The UPDB has been an important research population database for the discovery and elucidation of heritable factors for a wide variety of diseases, including cancer [2-6], influenza [11], asthma [12], diabetes [13], rotator cuff [14], aneurysm [15], and brain [16], among others. This resource and the high-risk pedigrees identified within it have been key for the localization and isolation of multiple cancer genes, including *CDKN2A *(melanoma) [17], *BRCA1 *[18], *BRCA2 *[19], and *HPC2/ELAC2 *(prostate cancer) [20].

The original Utah genealogy including 1.6 million Utah individuals has grown to include more than 6.5 million individual records, including 2.3 million individuals who belong to families of 3 to 15 generations deep, which connect to the original Utah genealogy. We have taken advantage of the extended genealogy data to include more rigorous control of the amount of ancestral genealogical data required for an individual to be included in genetic analyses. To ensure high quality genealogical records of sufficient depth and breadth for both cases and for controls, we only consider those individuals who have ancestral genealogy data for their parents, all four grandparents, and at least 6 of their 8 great grandparents. There were just over 1.2 million such individuals in the database used for this analysis. There are 85,550 of these 1.2 million individuals who also have a cancer record in the UCR between 1966 and 2009. We used ICD-oncology coding as indicated in Table 1 to assign these 85,550 individuals to 36 different cancer site groups.

**Table 1:** Definition of the cancer groups by ICD-O site and histology codes

Cancer	Site	Morphology	Behavior
Lip	0-9	8000-9589	2-9

Tongue	19-29	8000-9589	2-9

Salivary	79-89	8000-9589	2-9

Pharynx	90-119, 129-39, 140-48	8000-9589	2-9

Esophagus	150-159	8000-9589	2-9

Stomach	160-169	8000-9589	2-9

Small intestine	170-179	8000-9589	2-9

Colon	180, 182-189, 260	8000-8239, 8250-8719	2-9

Rectum	199, 209	8000-8239, 8250-8719	2-9

Anus	210-218	8000-9589	2-9

Liver/hepatic	220, 221	8000-9589	2-9

Gallbladder	239	8000-9589	2-9

Pancreas^α^	250-259	8000-1, 8010, 8140-1, 8480-1, 8500	2-9

Larynx	320-329	8000-9589	2-9

Lung/bronchus	340-349	8000-9589	2-9

Bone	400-419	8000-9589	2-9

Melanoma	0-809	8720-8790	2-9

Breast	500-509	8000-9589	2-9

Cervix	530-539	8000-9589	2-9

Endometrial^†^	540-549, 559	8010, 8050, 8140, 8210, 8260, 8310, 8323, 8380, 8382, 8441, 8460-1, 8480-81, 8560, 8570	2-9

Ovary	569	8000-9589	2-9

Female Genital (vulva, vagina and other unspecified)	510-519, 529, 570-79	8000-9589	2-9

Prostate	619	8000-9589	2-9

Testis	620-629	8000-9589	2-9

Bladder	670-679	8000-9589	2-9

Kidney	649, 659	8000-9589	2-9

Brain	710-719	8000-9529, 9540-9589	2-9

Thyroid	739	8000-9589	2-9

Multiple myeloma	0-809	9732	2-9

Chronic lymphocytic leukemia	0-809	8823	2-9

Acute lymphocytic leukemia	0-809	9821,9828,9831-7	2-9

Chronic myelocytic leukemia	0-809	9863, 9875, 9876	2-9

Acute myelocytic leukemia	0-809	9840,9861,9866-67, 9871-4, 9891, 9895-7, 9920	2-9

Non-Hodgkin's lymphoma	0-809	9530, 9590-1, 9596, 9598, 9670-1, 9673, 9675, 9678-80, 9684, 9687, 9689-91, 9695, 9698-702, 9705, 9708-9, 9714, 9716-9, 9727-29, 9755, 9764, 9827	2-9

Hodgkin's lymphoma	0-809	9650-5, 9659, 9661-7, 9945, 9946, 9948	2-9

Oropharynx	19, 24,51-52, 90-91,99,100-104, 108-9	8000-9589	2-9

^α ^Pancreatic adenocarcinoma only

^† ^Excluding endometrial sarcomas

Cancer rates in Utah are recognized to be generally lower than U.S. rates. Table 2 shows Utah/U.S. incidence rate ratios for cancers as defined and compared by SEER. Few cancer sites have significantly higher rates in Utah than the U.S. This is probably due to low rates of smoking in the Utah population [21].

**Table 2:** Cancer Incidence Rate Ratio: US/Utah rate ratio for US without Utah/Utah, by site, for males and females, 1995-2004, for those cancer sites with significantly higher, or significantly lower rates in Utah than the US

All Cancer Sites	Utah Lower	Utah Higher
Oral cavity and Pharynx	1.20 (1.19, 1.21)	

Esophagus	1.31 (1.24, 1.39)	

Stomach	1.66 (1.51, 1.82)	

Colon and Rectum	1.51 (1.41, 1.62)	

Anus	1.31 (1.27, 1.34)	

Liver and intrahepatic bile duct	1.23 (1.06, 1.44)	

Pancreas	1.89 (1.72, 2.07)	

Larynx	1.32 (1.25, 1.40)	

Lung and Bronchus	2.01 (1.80, 2.26)	

Melanoma (skin)		0.86 (0.83, 0.89)

Female breast	1.17 (1.14, 1.19)	

Cervix uteri	1.24 (1.15, 1.35)	

Corpus and uterus, NOS	1.08 (1.03, 1.13)	

Ovary	1.07 (1.01, 1.14)	

Prostate		0.95 (0.94, 0.97)

Testis		0.86 (0.79, 0.93)

Urinary bladder	1.25 (1.21, 1.30)	

Kidney and renal pelvis	1.33 (1.26, 1.40)	

Brain and other nervous system		0.94 (0.89, 0.99)

Thyroid		0.82 (0.78, 0.86)

Hodgkin's lymphoma	1.22 (1.11, 1.35)	

Non-Hodgkin's lymphoma	1.07 (1.03, 1.11)	

Myeloma	1.09 (1.02, 1.17)	

Leukemia	1.07 (1.03, 1.13)	

Rates are per 100,000 and age-adjusted to the 2000 US Standard population (19 age groups - Census p 25-1130) standard; Confidence intervals are 95% for rates and ratios [22,23].

### Genealogical Index of Familiality (GIF) method

The genealogical index of familiality (GIF) is a well-published method to test hypotheses of excess relatedness within sets of individuals selected from the UPDB [22,23]. The GIF test estimates the average pair-wise relatedness between all possible pairs within a defined group of individuals (cases) and compares it to the expected average relatedness in the UPDB. The average pairwise relatedness measured by the GIF is based upon the Malécot coefficient of kinship [24]; the coefficient gives the probability that randomly selected homologous genes from 2 individuals are identical by descent from a common ancestor. For parent-child relationships the Malécot coefficient is 1/2; for siblings or grandparent relationships the coefficient is ¼; for avunculars (e.g., aunt/niece or aunt/nephew) the coefficient is ⅛; for first cousins the coefficient is (1/2)^4^, or 1/16, and so forth. The value of the GIF statistic decreases with increasing genetic distance between individuals; unrelated individuals have 0 relatedness. All possible genetic paths between cases are identified and the average pairwise relatedness is summed and then averaged for all pairs, then multiplied by 10^5^.

The GIF statistic, or average pair-wise relatedness, for a set of cases is compared to GIF statistics estimated for 10,000 independent sets of matched controls; controls are matched by sex, 5 year birth cohorts, and birth state (Utah or not). The significance of the test of hypothesis is measured empirically based on the position of the case GIF in the distribution of 10,000 control GIF statistics. The overall GIF statistic, utilizing all pairwise relationships between all cases, allows a test of the alternative hypothesis of *no excess clustering *over that expected in the Utah population. A significant result for the Overall GIF test supports excess relatedness of the cases; however, it does not distinguish genetic from familial clustering resulting from shared environment or risk.

For this reason we have expanded the GIF method to include a comparison of average relatedness that ignores close relationships (with a relationship closer than first cousins). This revised "distant" GIF, or dGIF, statistic measures relatedness for distant relationships only, ignoring close relationships where shared environmental exposures are expected to be highest.

The dGIF statistic is calculated similarly to the original GIF, but ignores close relationships among cases, and among controls. A significant result for the dGIF test suggests an excess of *distant relationships *among case pairs and supports a genetic contribution to the observed excess relatedness.

For the GIF test, since we have performed multiple tests (on 36 different cancer sites), we have adjusted our threshold for significance using the Bonferroni correction (noted to be conservative). We use p < 0.0013 (0.05/36) to identify significant results.

The University of Utah Institutional Review Board, and the Resource for Genetic Epidemiological Research approved the research.

## Results

The GIF test for excess relatedness was performed for the 36 different cancer groups shown in Table 1. Even though we have increased our stringency on the cases to be analyzed (with respect to the amount of ancestral genealogy data available) sample sizes have still increased substantially from the 1994 analysis e.g., prostate cancer increased from 8,060 cases in 1994 to 13,933 cases in this study. Nine cancer sites previously not analyzed due to inadequate sample size in 1994 are presented here for the first time. These newly analyzed cancer sites include: larynx (n = 427), female genitals (n = 365), anus (n = 396), tongue (n = 347), pharynx (n = 315), oropharynx (n = 261), salivary (n = 338), esophagus (n = 520), and bone cancers (n = 294).

Results of the GIF test, shown in Table 3 include the cancer site, the number of cases, the overall case average relatedness (case GIF), the average relatedness of controls (mean control GIF), the overall average distant relatedness (case dGIF), the average distant relatedness of controls (mean control dGIF), the standard deviation of the control GIF (SD Control GIF), and the standard deviation of the control dGIF (SD Control dGIF), the empirical significance for overall excess of relatedness, and the empirical significance for the distant relatedness (dGIF) statistic. The empirical significance for the GIF test of overall familial clustering (both close and distant) is the same algorithm used to calculate the GIF in previously published reports on the familiality of cancer in Utah [3,4]. The GIF values for both cases and controls are higher now than in previous publications for two reasons, we are analyzing more recent birth cohorts and we are only considering those individuals with specific ancestral genealogy requirements.

**Table 3:** Genealogical Index of Familiality for cancers with at least 200 cases with genealogy data, ranked by empirical significance for overall excess relatedness (and ranked by case GIF where the overall empirical significance values are equivalent)

Cancer	N	Case GIF	Mean Control GIF	Case dGIF	Mean Control dGIF	SD Control GIF	SD Control dGIF	Empirical p GIF	Empirical p dGIF
Chronic Lymphocytic Leukemia	978	7.90	4.76	5.10	4.14	0.26	0.20	< 0.0001	< 0.0001

Small Intestine	387	7.49	4.67	4.56	4.12	0.53	0.38	< 0.0001	0.137

Lip	1077	7.26	4.83	4.89	4.14	0.25	0.20	< 0.0001	0.0001

Thyroid	1865	6.38	4.49	4.48	4.07	0.13	0.11	< 0.0001	0.0006

Testis	691	5.97	4.37	4.18	4.01	0.26	0.20	< 0.0001	0.1943

Multiple Myeloma	1268	5.90	4.76	4.59	4.14	0.21	0.17	< 0.0001	0.0067

Prostate	18069	5.57	4.76	4.44	4.15	0.03	0.03	< 0.0001	< 0.0001

Melanoma	4463	5.56	4.57	4.53	4.11	0.08	0.07	< 0.0001	< 0.0001

Colon	6285	5.49	4.78	4.26	4.13	0.07	0.06	< 0.0001	0.0233

Lung/Bronchus	5063	5.42	4.75	4.27	4.14	0.08	0.07	< 0.0001	0.046

Rectum	2472	5.38	4.73	4.34	4.13	0.13	0.11	< 0.0001	0.032

Kidney	1791	5.35	4.65	4.36	4.12	0.15	0.13	< 0.0001	0.035

Ovary	1941	5.32	4.68	4.27	4.11	0.14	0.12	< 0.0001	0.1042

Non Hodgkin Lymphoma	3267	5.26	4.65	4.44	4.12	0.10	0.08	< 0.0001	0.0002

Breast	12523	5.10	4.70	4.16	4.12	0.04	0.04	< 0.0001	0.2009

Stomach	1490	5.66	4.86	4.32	4.16	0.19	0.16	0.0001	0.1553

Pancreas	1893	5.45	4.77	4.32	4.13	0.16	0.13	0.0001	0.0757

Bladder	2455	5.29	4.84	4.15	4.14	0.13	0.11	0.0008	0.4479

Hodgkin Lymphoma	752	5.25	4.44	3.88	4.03	0.26	0.20	0.0018	0.7628

Endometrial	3155	5.04	4.72	4.03	4.12	0.11	0.09	0.002	0.8261

Cervix	922	5.35	4.68	4.20	4.12	0.26	0.20	0.007	0.3315

Larynx	427	6.06	4.76	4.41	4.14	0.52	0.38	0.0121	0.2366

Brain	1488	4.83	4.45	4.03	4.03	0.16	0.13	0.0124	0.5067

Anus	396	6.33	4.98	4.81	4.17	0.62	0.43	0.0226	0.079

Genital Female	365	6.01	4.75	4.32	4.12	0.60	0.43	0.0266	0.3117

Salivary	338	5.95	4.68	4.52	4.13	0.61	0.44	0.0286	0.1808

Liver/Hepatic	449	5.50	4.63	4.32	4.10	0.47	0.34	0.0418	0.253

Pharynx	315	5.82	4.64	3.30	4.10	0.65	0.46	0.0447	0.9682

Acute Myelogenous Leukemia	920	5.01	4.62	3.84	4.09	0.25	0.19	0.0651	0.8933

Tongue	347	5.57	4.65	3.49	4.13	0.58	0.42	0.0686	0.9418

Acute Lymphocytic Leukemia	409	4.58	4.14	3.98	3.83	0.38	0.27	0.1284	0.2822

Gallbladder	306	5.49	4.90	4.15	4.15	0.77	0.53	0.2106	0.4799

Esophagus	520	4.83	4.71	3.90	4.15	0.42	0.31	0.3814	0.7854

Chronic Myelogenous Leukemia	343	4.59	4.69	4.16	4.10	0.61	0.44	0.5345	0.4319

Oropharynx	261	4.48	4.68	3.37	4.13	0.77	0.54	0.5686	0.9274

Bone	294	3.66	4.34	3.66	3.97	0.56	0.40	0.9014	0.7829

### Distant genealogical index of familiality results

We also present, for the first time for cancer, our expansion of the GIF test to include a test for excess distant relatedness (dGIF). The case, control dGIF statistics, and the empirical significance for the dGIF test are shown for all cancer sites in Table 3. This analysis identifies 6 cancer sites for which a significant excess relatedness is observed in distant relationships (p < 0.0013, corrected), strongly supporting a genetic contribution to cancer predisposition. These 6 cancers include some with well known genetic effects and already identified predisposition genes, as well as some for which a genetic hypothesis has not yet been made. The 6 sites include chronic lymphocytic leukemia, thyroid, lip, lung, prostate, and melanoma.

We note that some cancers with already identified predisposition genes (e.g. breast cancer, colon cancer) did show significant evidence for excess relatedness, but did not show significant excess familiality under the dGIF test. Multiple gene identifications of subsets of breast and colon cancers have provided clear evidence of a genetic contribution to some portion of these two cancer sites (considered independently), but it is noted that the percent of familial cases explained by the known predisposition genes is low, and that the known predisposition genes primarily explain dense pedigrees with close relatives. It therefore may not be a surprise that the dGIF test shows borderline significance for colon cancer and is not significant for breast cancer.

We consider the dGIF a conservative test for this reason. Those cancers identified to have a significant dGIF test represent those cancers with the most significant evidence for a genetic predisposition, exhibiting excess risk in distant, as well as in close relatives. However, the dGIF test is not the only indication for a genetic contribution to predisposition. Typically, when analyzing a single cancer site, we use both Relative Risk estimates and GIF tests to more completely test the hypothesis of a genetic contribution.

Similar to previously published GIF analyses [8,12], we observed a significant excess of relatedness of cases for most cancers studied. Before correction for multiple testing, all but 8 of the cancers considered show significant overall excess clustering of cases. The 8 sites not showing a significant excess even when considered independently all have small samples sizes (261 ≤ n ≤ 920) and many have recognized environmental risk factors (e.g., esophageal cancer and smoking). Some of the cancers with a non-significant overall p-value had borderline significant excess (e.g. AML and tongue cancer) and some cancer sites had a much higher case GIF than matched control GIF (e.g. gallbladder cases = 5.49 and controls = 4.90). Larger sample sizes in future years may clarify these results.

The GIF statistic values for cancer cases range from 3.66 for bone cancer to 7.90 for chronic lymphocytic leukemia (this subgroup of leukemia cases was also one of the highest GIF measures observed in the 1994 analysis). Mean GIF statistics for the 10,000 sets of matched controls for each site had a smaller range (4.14 - 4.98); this is as expected in light of the overall similarity we expect for the estimated pairwise relatedness of randomly selected UPDB controls matched to cancer cases for 5-year birth cohort and sex. The control GIF measure represents the average expected relatedness in the Utah population for older individuals (those representing the at-risk cancer population). There is also some expected variability of the control GIF values based on the birth year distributions for different cancer sites. Individuals in the UPDB with earlier birth years have lower relatedness than those born more recently. The lowest control GIF average (4.14) was for acute lymphocytic leukemia; only 20 of 409 ALL cases were born before 1900; the highest control GIF statistic (4.98) was for cancer of the anus, 117 of the 396 anus cancer patients were born before 1900.

## Discussion

This analysis of cancer familial clustering provides a comprehensive review of evidence for familial clustering of cancer by site using a population-based genealogical resource linked to statewide cancer data. This analysis serves to confirm previously reported conclusions suggesting that the majority of cancer sites show some evidence of familial clustering in excess of expected. The only cancer sites not showing overall significant excess familial clustering are those with the smaller sample sizes, suggesting that it may be prudent to await more data analysis before a conclusion is reached for these rarer cancers. Using a more stringent, conservative test for a genetic contribution to cancer predisposition (the dGIF), this analysis has identified some cancer sites with significant evidence for a genetic contribution to predisposition, including very strong evidence for chronic lymphocytic leukemia, thyroid cancer, lip cancer, lung cancer, prostate cancer, and melanoma.

In addition to adding multiple new cancer sites to this analysis, and multiple new subgroups of cancers, we additionally used a more stringent selection criteria for cases and controls, based on amount and quality of genealogical data available. We propose that this increases the fidelity of the results and serves to eliminate the noise in the genealogical resource that comes from analysis of a greater number of families with incomplete genealogy compared to analysis of individuals known to have multiple relatives whose cancer can be observed in our window of view from 1966. In addition, this filtering based on quantity of ancestral genealogy has resulted in slightly higher case GIF statistics than were observed in, for example, the 1994 analysis.

The results for the overall GIF test show that for most of the cancer sites examined (28 of 36) there is a significant excess overall relatedness observed (without correction). This is similar to the results shown in Cannon-Albright et al., 1994, the most recent published Utah GIF analysis. In that previous analysis, all cancer sites except small intestine, gallbladder, kidney, liver, pancreas, and uterus (termed endometrial in this analysis) showed significant overall excess relatedness (at p < 0.05). With larger sample sizes, we now see significant excess cancer clustering of the small intestine, kidney, liver, pancreas, and uterus (endometrial). Of the new sites we have added to this analysis, larynx, anus, salivary, pharynx and female genitals show significant overall excess familial clustering. The list of cancers that failed to show excess overall familial clustering primarily includes those with the smallest sample sizes. Separate analyses of cancers have identified subgroups with evidence for a genetic contribution [16,25-27].

The familiality analyses reported here has been limited in terms of the availability of data. Cancers diagnosed before 1966 or outside of Utah are censored; similarly individuals whose genealogy data was not included in the UPDB, or whose data did not appropriately link to their cancer data are also censored. We assume such censoring to be unbiased in nature. Because the GIF analysis considers relationships that are observed, it is robust to such censoring, but may be conservative in its identification of strong evidence for a heritable predisposition to disease.

Increases in computing power have allowed us to increase the number of matched controls groups analyzed from n = 6 in 1982, to 100 in 1994, to 10,000 in this analysis. It has similarly allowed us to consider both overall excess relatedness, as well as excess relatedness due only to distant relationships, the key to being able to separate what could be clustering due to shared environment from clustering that appears much more likely to be due to shared genetic factors.

## Conclusions

Although we report several cancer sites with strong evidence for a genetic contribution, the implications of the results reported here go beyond genetic predisposition to disease. For almost all cancer sites analyzed, we observed an excess of familial clustering; most of this evidence is based on an excess of close relationships among cases. Whether based on the existence of shared environment or of shared genetics or a combination, the clinical implications are the same: close relatives of individuals with cancer are at increased risk for cancer of that same site. This is true for cancers of many different sites, and this should be considered when making decisions on cancer screening or recommendations for lifestyle changes.

The implications for those cancers with evidence for both close and distant excess relatedness are also clear. There must exist genes or gene variants that are responsible for the clustering observed in the Utah population. Multiple cancer predisposition genes have already been identified in high-risk Utah pedigrees identified in the UPDB, including *BRCA1, BRCA2*, and *p16/CDKN2A *[17-19]. The newly reported cancers of most interest for a genetic contribution to predisposition (lip cancer, non-Hodgkin lymphoma and multiple myeloma) should be the focus of high-risk pedigree studies to identify the hypothesized predisposition genes for which we present evidence. Such studies have begun in Utah, and our findings may have implications for cancer screening and risk assessment.

## Competing interests

The authors declare that they have no competing interests.

## Authors' contributions

FA performed all analyses and produced the primary manuscript. CT and TLW participated in discussions of appropriate groups for analysis. LACA conceived of the study and provided oversight. All authors contributed to, read, and approved the final manuscript.

## Pre-publication history

The pre-publication history for this paper can be accessed here:

http://www.biomedcentral.com/1471-2407/12/138/prepub
